# 2765. Dalbavancin for challenging left ventricular assist device driveline infections

**DOI:** 10.1093/ofid/ofad500.2376

**Published:** 2023-11-27

**Authors:** Christina Doligalski, Anne Lachiewicz, Anne Friedland, Cynthia Snider, Amanda Bowen, Ashley H Marx, Mirnela Byku, Luther A Bartelt

**Affiliations:** UNC Health Department of Pharmacy, Chapel Hill, North Carolina; University of North Carolina, Chapel Hill, NC; UNC School of Medicine, Chapel Hill, North Carolina; Cone Health, greensboro, North Carolina; UNC Health, Chapel Hill, North Carolina; University of North Carolina Medical Center, Chapel Hill, North Carolina; UNC School of Medicine, Chapel Hill, North Carolina; University of North Carolina School of Medicine, Chapel Hill, NC

## Abstract

**Background:**

Driveline tract and exit site infections (DLI) in patients on left ventricular assist device (LVAD) support occur frequently and are associated with significant morbidity, mortality and recurrence. Dalbavancin is a gram+ active lipoglycopeptide antibiotic with a prolonged half-life. Given a variety of scenarios including prior antibiotic failure, interacting concurrent therapies and socioeconomic factors, dalbavancin may be of interest for use in LVADs with challenging gram+ DLIs.

Case Series Details

**Figure:**
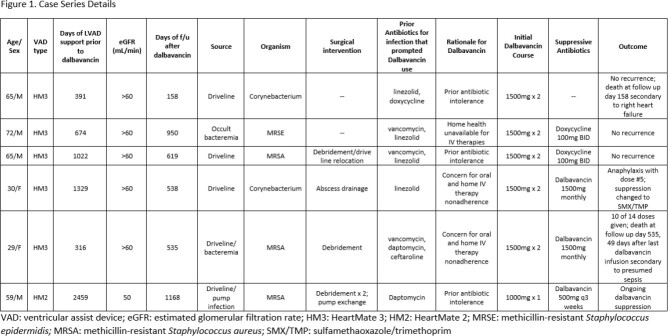

**Methods:**

A chart review was conducted for all LVADs at a single center who received at least one dose of dalbavancin prior to 1/1/2023 to assess safety and to describe utilization. Demographic data, microbiologic and antibiotic histories, and dalbavancin therapies along with further suppressive antibiotics were recorded.

**Results:**

Six patients on LVAD support received dalbavancin for different gram+ infections (Figure 1). Five of the six had confirmed DLI. Three patients received two doses of dalbavancin 1500mg 1-2 weeks apart, followed by suppressive doxycycline with no infection recurrence to date. Three patients received dalbavancin initial doses 1-2 weeks apart followed by planned suppressive dalbavancin at 3-4 week intervals. One of these patients developed anaphylaxis with dose #5 and was subsequently changed to suppressive sulfamethoxazole/trimethoprim. Another received 10 of 14 planned doses over a 14 month period, with controlled infection while adherent to infusion. The final patient remains on suppressive dalbavancin ( >1100 days). Apart from the anaphylactic reaction, no other adverse events were documented related to dalbavancin therapy.

**Conclusion:**

In our limited case series, adherence with dalbavancin was a successful therapeutic option for patients with LVAD-related gram+ infections after failure of other therapies or socioeconomic limitations. Anaphylaxis was observed in one patient during infusion #5, suggesting the need for ongoing monitoring despite prior tolerability. One patient remains on prolonged suppressive intermittent dalbavancin, suggesting extended use may be viable in highly select patient scenarios. Further data is needed on prolonged durations and optimal dosing strategies in complex device-related infections.

**Disclosures:**

**Anne Lachiewicz, MD, MPH**, Cidara: Grant/Research Support|Contrafect: Grant/Research Support|Novartis: Grant/Research Support|Pfizer: Grant/Research Support|Shiongi: Grant/Research Support **Mirnela Byku, MD**, Abbott: Advisor/Consultant

